# Preserving Motor Features by Alternative Re‐Referencing to Remove Heart Artifact on the Stentrode

**DOI:** 10.1002/advs.76647

**Published:** 2026-09-04

**Authors:** Ariel K. Feldman, Kriti Kacker, Rahyun Yun, Nikole Chetty, James Bennett, Peter E. Yoo, Raul G. Nogueira, Jennifer L. Collinger, Nicholas L. Opie, Thomas J. Oxley, David F. Putrino, Douglas J. Weber

**Affiliations:** ^1^ Neuroscience Institute Carnegie Mellon University Pittsburgh Pennsylvania USA; ^2^ Center for the Neural Basis of Cognition Pittsburgh Pennsylvania USA; ^3^ Department of Mechanical Engineering Carnegie Mellon University Pittsburgh Pennsylvania USA; ^4^ Department of Electrical & Computer Engineering Carnegie Mellon University Pittsburgh Pennsylvania USA; ^5^ Vascular Bionics Laboratory, Department of Medicine, Royal Melbourne Hospital University of Melbourne Parkville Victoria Australia; ^6^ Synchron, Inc. New York USA; ^7^ Department of Neurology and Neurosurgery, University of Pittsburgh Medical Center Stroke Institute Pittsburgh Pennsylvania USA; ^8^ Rehab Neural Engineering Labs University of Pittsburgh Pittsburgh Pennsylvania USA; ^9^ Department of Physical Medicine and Rehabilitation University of Pittsburgh Pittsburgh Pennsylvania USA; ^10^ Department of Bioengineering University of Pittsburgh Pittsburgh Pennsylvania USA; ^11^ Department of Biomedical Engineering Carnegie Mellon University Pittsburgh Pennsylvania USA; ^12^ Department of Rehabilitation and Human Performance Icahn School of Medicine at Mount Sinai New York USA

**Keywords:** brain‐computer interface, endovascular, spatial filtering, stentrode

## Abstract

Vascular electrocorticography (vECoG) has shown great promise as a less‐invasive alternative to penetrating electrode arrays for neural signal acquisition. This study investigates the signal quality of the Stentrode device, a leading vECoG platform, by contrasting artifact persistence with the preservation of low frequency motor cortical activity. Typical (re‐)referencing schemes, including a monopolar stent‐mounted reference, a common average reference, and a Laplacian reference, are shown to significantly reduce the presence of electrocardiogram (ECG) artifacts compared to a distal monopolar reference. However, resting state beta activity is also significantly diminished when employing these techniques. By using Band‐Limited Independent Component Analysis (BL‐ICA), a type of spatial filter that allows weighting of the noise on each electrode differently, ECG artifacts are easily separated from vECoG recordings. With this cleaning methodology, signals are reconstructed without the ECG component and the reduction in cross‐channel correlation is evaluated, as well as the increase in relative entropy between rest and go distributions. To ensure that low‐frequency motor features are preserved, beta bursts features are evaluated in both the source space and reconstructed signal space. BL‐ICA is an effective technique to remove widespread ECG artifacts while maintaining typical motor‐related features in beta band activity.

## Graphical abstract

Endovascular brain‐computer interfaces record neural activity from within cerebrovasculature, at the expense of electrocardiogram contamination. Band‐limited independent component analysis separates this heart‐based artifact from task‐relevant neural activity in each frequency band, enabling the reconstruction of cleaner neural recordings without the signal loss caused by other re‐referencing measures typically employed on the Stentrode.

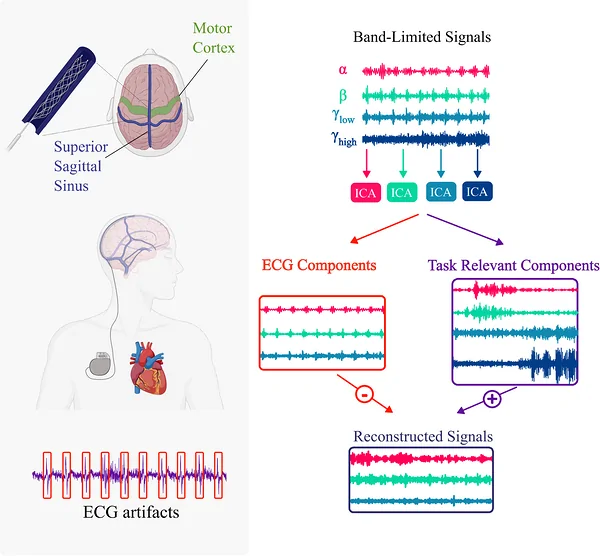

## 1 Introduction

Bridging the gap between the mind and the external environment, brain‐computer interfaces (BCIs) hold great promise for enabling independence in patients with severe motor deficits. These devices, with capabilities ranging from controlling a robotic arm [1, 2, 3, 4] to real‐time speech production [5, 6, 7, 8, 9], are rapidly evolving, with recent years witnessing significant advancements in the creation and commercialization of novel BCIs [10, 11, 12]. The potential impact of these devices is expansive, yet their utility in practice is contingent on the quality of the signals they capture and interpret.

To ensure the reliability and effectiveness in a home setting, the signal quality of any BCI must be thoroughly investigated and characterized. BCIs involve recording modalities, ranging from single‐unit recordings to population‐level neuronal activity. The signal resolution achieved by a BCI depends largely on the category of the interface, which generally falls into one of three classifications: non‐invasive, less‐invasive, and invasive [13]. This classification is primarily based on the deployment technique, channel density and the associated risks.

Among the most widely used non‐invasive BCI modalities is electroencephalography (EEG), which uses a cap with electrodes that contact the scalp through a conductive gel. EEG offers a spatial resolution of approximately 10 mm and a temporal resolution of around 50 ms [14]. However, since EEG signals must traverse multiple biological layers—including the scalp, skull, tissue, and cerebrospinal fluid—before being recorded, they are susceptible to attenuation and noise. The electrode‐tissue interface introduces both resistive and capacitive properties, resulting in a low‐pass filtering effect that attenuates higher‐frequency components of the signal [14]. Consequently, EEG captures local field potentials, with analysis primarily focused on lower‐frequency bands such as alpha and beta.

Invasive BCI technologies include subdural and epidural electrocorticography (ECoG), which record neural activity from populations of neurons, as well as intracortical microelectrodes that capture both single‐unit and multi‐unit activity. These methods offer significantly higher resolution than non‐invasive approaches, with spatial resolution ranging from approximately 1 mm to 0.05 mm and temporal resolution up to 3 ms [15]. As of 2024, 67 participants globally have participated in clinical trials to study the safety of implantable BCIs [16].

Recent advancements in less‐invasive bioelectronic devices aim to bridge the gap between non‐invasive and invasive approaches by providing access to neural signals without open brain surgery and the associated postoperative complications. These efforts include magnetoelectric‐material‐based wireless neural stimulators tested in animal models and humans [17, 18], endocisternal interfaces tested in sheep models [19], simulated transnasal electrode placement [20, 21], and endovascular stent‐electrode arrays [22] tested in both sheep models and through long‐term implantation in humans [23, 24].

ECG artifacts have long marred the integrity of observed neural signals, from EEG to magnetoencephalography (MEG) recordings [25, 26, 27]. Typically altering the signal in the range of 0.5–40 Hz, these artifacts affect the content of a majority of the cortical bands of interest [28]. Their impact bleeds into the power spectra of a signal, often visible when computing the power spectral density (PSD) and likely altering the aperiodic component of neural signals [27, 29]. Previous analysis of combined cardiac and neural data suggest that the frequency domain is strongly influenced by the ECG artifact, even when it is not prominent in the time domain [27].

The Stentrode (Synchron, Inc., Brooklyn, New York, USA) is a less‐invasive endovascular electrode‐array BCI that is implanted via the jugular vein and positioned adjacent to the motor cortex [23]. Seated in the Superior Sagittal Sinus (SSS), the Stentrode is constantly exposed to the rhythmic flow of blood through the vascular system, which can lead to intense electrocardiogram (ECG) artifacts on vECoG recordings — thus, proper methods to separate motor cortical signals from ECG noise are necessary. Due to the novel nature of vECoG recordings, it is not yet understood how to perform this separation cleanly. Previously, Independent Component Analysis (ICA) was explored as a technique to perform this separation on broadband (0.5‐250 Hz) vECoG data. ICA has been demonstrated as an effective technique to remove heart artifacts in EEG and MEG signals [30, 31, 32]. While ICA demonstrated a greater reduction in ECG artifacts compared to offline monopolar re‐referencing (see Section 2.4), the resultant signals exhibited less differentiation in the gamma bands than expected [33].

Referencing schemes substantially affect the features present in recorded signals [34, 35, 36]. Poor reference selection can completely obscure features of the signal [37]. The present study attempts to devise a better referencing scheme for vECoG data by optimizing for the following parameters: reduce the influence of ECG artifact, improve the signal‐to‐noise ratio (SNR) of motor cortical signals, and preserve the motor features of the neural signals captured. In this vein, we explore common re‐referencing schemes as well as a modified blind source separation technique to enhance the signal quality of Stentrode vECoG recordings.

## 2 Materials & Methods

### 2.1 Participants

The analysis in this paper focuses on the neural recordings from 5 participants (referred to as P1‐P6) in the COMMAND EFS clinical trial (NCT05035823). While there were 6 participants in the COMMAND EFS clinical trial, one participant (P2) was excluded from most analyses as no motor data was collected from them with the distal reference scheme (see Section 2.4). Their baseline recordings with a distal reference scheme, however, were included. This study was conducted under an Investigational Device Exemption from the U.S. Food and Drug Administration and approved by the Institutional Review Boards at Western‐Copernicus Group, Mount Sinai Hospital, University of Pittsburgh, University of Buffalo, and Carnegie Mellon University. Informed consent was obtained before any study procedures were conducted.

All participants had severe paralysis due to Amyotrophic Lateral Sclerosis (ALS) except P4 (44F), who had severe upper‐limb paralysis due to an acute ischemic stroke in the brainstem. P1, P3 and P5 (67 M, 55 M, 53F) had full‐body paralysis, resulting in no voluntary movement of the extremities, an inability to communicate verbally, and ventilator‐dependence from severely diminished respiratory function. P6 (63 M) had Flail Arm Syndrome, a subset of ALS that was characterized by the onset of weakness isolated to the arms before progressing to the rest of the body [38, 39, 40]. At the time of implant, P6 was able to walk and talk, but had little motor function remaining in his arms.

### 2.2 Device and System Details

**FIGURE 1:**
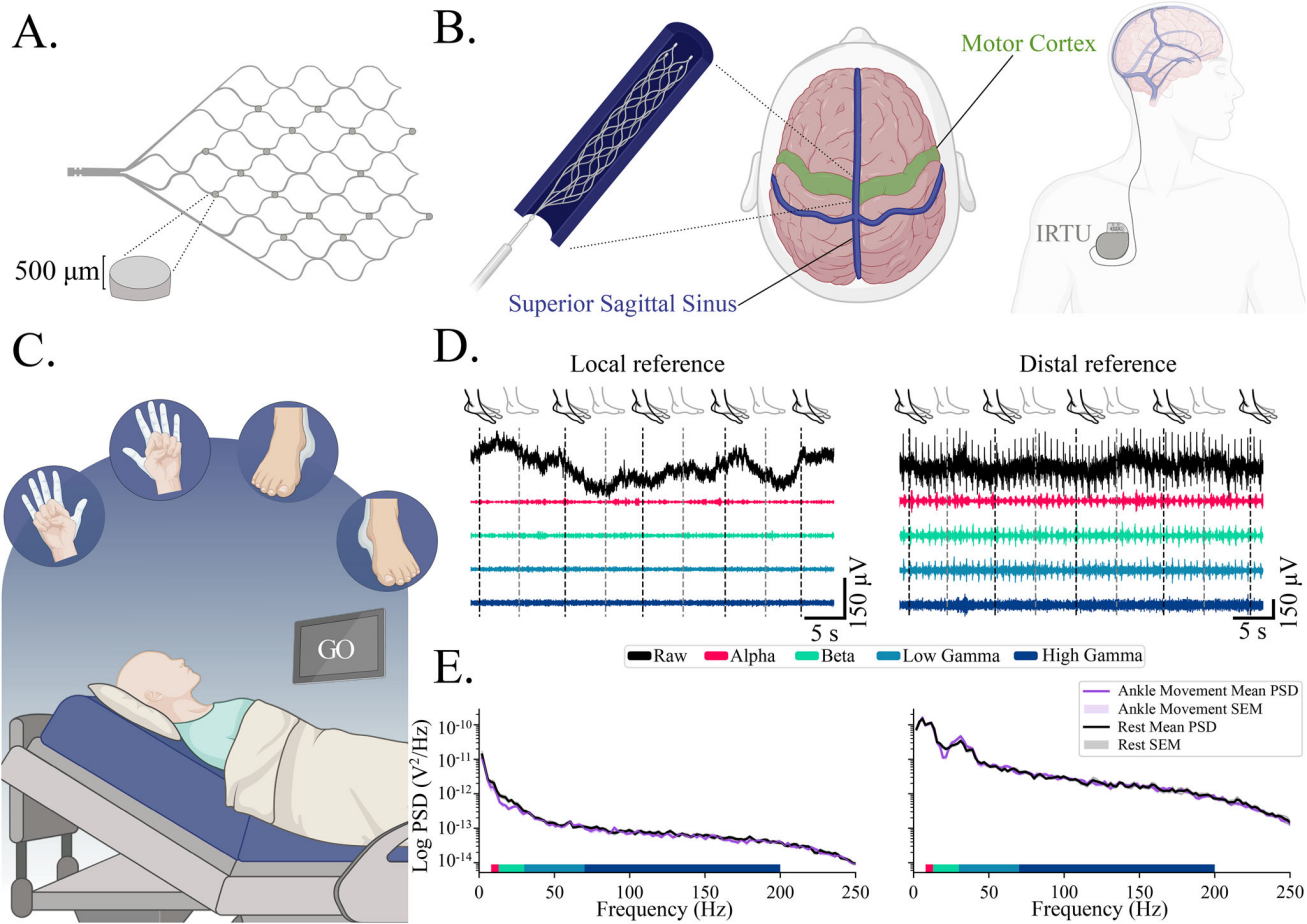
Experimental design and sample vECoG recordings. Representation of the Stentrode, the motor mapping task and example recorded vECoG signals. (A) Diagram of the unfurled Stentrode, showing the placement of 16 electrodes along the scaffolding of the stent. (B) Diagram of Stentrode placement in the cerebral venous system, as well as the lead and the implantable receiver transmitter unit (IRTU). The green highlights the bilateral motor cortices. The blue represents the major cerebral veins, including the Superior Sagittal Sinus (rostral‐caudal) and the Superior Anastomotic Vein (medial‐lateral). The IRTU is seated in the chest of a participant and the lead connects it to the Stentrode. (C) Diagram of the motor mapping task the participants performed throughout this study. Participants are either seated or reclined during the task, and they are instructed which movement to execute before the run begins. (D) Sample recordings from channel 3 of P6 during the motor mapping task when right ankle movement is being performed with either the local or distal reference being used. (E) Log power spectral density of the channel illustrated in 1D, averaged over the 10 trials of rest and 10 trials of ankle movement in one run. The average PSD as well as the standard error are plotted, with color bars on the x‐axis demarcating the bounds of each cortical frequency band.

The Stentrode comprises a self‐expanding Nitinol stent. 16 platinum electrodes, with an interelectrode distance of approximately 3 mm [23] and a diameter of 500 µm, were mounted on the stent (Stentrode, Synchron, CA, USA) [24] (Figure 1A). Vascular electrocorticographic (vECoG) data recorded by the Stentrode was sampled at 2 kHz, with a hardware low pass filter at 500 Hz. Of the 16 stent‐mounted electrodes, only those with an impedance below 100 kΩ were included in the analyses for this paper (Table 1).

**TABLE 1:** Participant details. For each of the 6 participants whose data are analyzed in this paper, we list their gender, age at time of implantation, cause of paralysis, the number of stent‐mounted channels used for further analysis, and the durations of the go and rest periods in the format mean (standard deviation) in seconds.

Participant ID	Gender	Age	Cause of paralysis	Channels used	Go period duration (s)	Rest period duration (s)
P1	Male	67	Amyotrophic Lateral Sclerosis	13	4.999 (0.020)	5.025 (0.558)
P2	Male	73	Amyotrophic Lateral Sclerosis	9	N/A	N/A
P3	Male	55	Amyotrophic Lateral Sclerosis	12	5.008 (0.015)	5.168 (0.433)
P4	Female	44	Acute Ischemic Stroke in brainstem	11	4.985 (0.048)	5.241 (0.511)
P5	Female	53	Amyotrophic Lateral Sclerosis	10	4.990 (0.030)	4.972 (0.570)
P6	Male	63	Amyotrophic Lateral Sclerosis	9	4.411 (1.398)	4.964 (0.591)

Pre‐implantation structural computed tomography and functional magnetic resonance imaging (fMRI) was performed to verify the presence of detectable neural activity in the primary motor cortices (M1) along the SSS. To assess the latter, participants were asked to attempt flexion and extension of their ankles during the fMRI. Once discernible activity was confirmed, a catheter guided the Stentrode through the jugular vein to the SSS. The Stentrode was deployed between the M1 cortices, and full expansion was confirmed via contrast‐enhanced angiography.

From the Stentrode, a transvascular lead carried the recorded vECoG signals to the implantable receiver transmitter unit (IRTU, Synchron, NY, USA) in a subcutaneous pocket in the chest. An external telemetry unit (ETU) was laid on the participant's chest, over the IRTU, for wireless transmission of the vECoG signals. The surgical procedures and signal acquisition methods have been comprehensively outlined in prior studies [23, 24].

### 2.3 Task Design

Neural data was acquired while participants performed a motor mapping task (Figure 1C). When prompted by a screen, participants were asked to attempt one of 6 movement types 5 times within a 5‐second “go” period. Each go period was followed by a rest period lasting approximately 5 seconds (Table 1). A run consisted of 10 go periods and 10 rest periods for a single movement. A completed run would therefore contain 50 attempted movements. The 6 movement types were as follows: left hand, right hand, bilateral hand, left ankle, right ankle, and bilateral ankle. For the hand movements, participants were instructed to attempt to make a fist with the specified hand(s) and release, as cued by the visual on the screen. For the ankle movements, participants were instructed to extend their ankle(s) as if they were pressing on a gas pedal, and similarly release with the cued visuals. During rest periods, participants were instructed to relax their limbs.

Additionally, at the start of each session, a baseline run was recorded during which the participant was instructed to sit still and relax their muscles for two minutes. These baseline recordings were performed both with the distal and local hardware references. A more detailed explanation of the referencing schemes can be found in 2.4.

### 2.4 Referencing

Two main referencing schemes have been used throughout the COMMAND EFS clinical trial: a monopolar distal reference and a monopolar local reference. The distal reference subtracted a channel located in the IRTU, positioned in the chest cavity, from all stent‐mounted channels. The local reference was selected along the stent body by determining which of the implanted, stent‐mounted channels yields the best signal‐to‐noise ratio. For the ECG artifact analysis performed in Section 3.1, the local reference PSDs were computed offline on the distally referenced signals to compare the amplitudes of the same ECG artifacts.

Beyond these two hardware references, we also evaluated the impact of several re‐referencing schemes on the distally referenced data. The first scheme was the Common Average Reference (CAR), which computes the average signal from all channels with impedance less than 100 KΩ and subtracts it from each stent‐mounted channel individually. This re‐referencing scheme has been applied to the Stentrode in previous studies [41, 42, 43]. The second scheme was Laplacian re‐referencing, which performs a more localized version of the CAR by removing the average of the two to three nearest electrodes around each contact.

After re‐referencing the signals, Wilcoxon Signed‐Rank Tests were used to compare the artifact peak‐to‐peak amplitude (µV) in the distally referenced data to that of the local, CAR and Laplacian re‐referenced data.

### 2.5 VECoG Analysis

A second‐order infinite impulse response notch filter was applied at 60 and 120 Hz to eliminate line noise and its harmonics. For re‐referenced data, the notch filtering was applied after re‐referencing. All analysis was performed offline in Python.

The power spectral density (PSD) of each run was computed for both the rest and go periods of the task using Welch's method [44]. To calculate the PSDs, a window size of 512 ms and an overlap of 50% were used. Two PSDs were calculated for each channel – one over each rest period and one over each go period. For each condition on a channel, the PSDs were averaged and the standard error was calculated (Figure 1E). Additionally, the PSD for each channel during each baseline recording was calculated. We then computed the average and standard deviation for each referencing scheme. Using FOOOF [45], we parameterize the average PSD for each referencing scheme to estimate the oscillatory and aperiodic components of the signal at rest. Oscillatory and aperiodic components were fit over a 1–200 Hz frequency range with peak width limits of 3.0–25.0 Hz and a peak threshold of 2.0 standard deviations. By subtracting the aperiodic component, also called the noise floor, from the average PSD, we can calculate the oscillatory component of the PSD – the power of any frequency over the noise floor. Over the canonical beta band boundaries (13–30 Hz), we can calculate the oscillatory beta power (d_β_) as the mean difference between the average PSD and the fitted aperiodic component in µV^2^/Hz.

For the band‐limited analyses, this data was then band‐passed into alpha (8‐13 Hz), beta (13‐30 Hz), low gamma (30‐70 Hz) and high gamma (70–200 Hz) [46] bands using a fifth‐order Butterworth filter.

For the power difference analysis, we calculate the average power for each rest ($P_{rest}$) and go ($P_{go}$) period. A delay of 250 ms was added to each cue presentation when extracting the rest and go periods to account for the participant's reaction time. The power was calculated as the average of the squared signal amplitudes for a trial. This value was then converted to decibels. For each go period, we then calculate the difference with the preceding rest period.

To assess the envelope of the band‐passed signals, the analytic signal of each trial was computed via the Hilbert transform. The absolute value of this analytic signal was then used to extract the envelope of the signal.

### 2.6 Independent Component Analysis

Independent Component Analysis (ICA) was used to decompose the neural signals collected by the Stentrode into source space signals using the FastICA algorithm [47]. First, ICA was fit to broadband data, and the resulting independent components were inspected visually for electrocardiogram (ECG) artifact. Then, after band‐pass filtering the original vECoG signals, the channels for each band were scaled to unit variance and ICA was applied to each frequency band separately – a procedure we will refer to henceforth as band‐limited ICA (BL‐ICA). Thus, 4 unmixing matrices were constructed for each run of 10 motor mapping trials.

However, the number of components used in the ICA decomposition greatly affects the estimated source space signals. For each run of the motor mapping task, an ICA unmixing matrix was found for each cortical frequency band. Once in the source space, we reconstruct the data in the signal space using the tuned mixing matrix – this weights the source space signals to recreate the observed signals as closely as possible. Reconstruction error was then calculated for each channel as the difference between the observed signal and the reconstructed signal. Further penalizing errors in the linear transformation built by ICA, we calculate the L2 norm of the reconstruction error. The median of the L2 norms across channels was then recorded as the error for a run. This was repeated at each possible number of components, which represents the number of sources to find, for all distally referenced runs (Figure S1).

After determining the appropriate number of components, the ECG artifact was identified visually among the band‐limited independent components (BL‐ICs) in the source space. Potential ECG artifacts in the BL‐ICs were identified using BioSPPy [48] to quantify the R‐R intervals and compute the template waveform of the band‐limited artifacts. BL‐ICs were flagged for removal if the R‐R distribution was in the physiological range for a participant, if the detected heart rate in the potential ECG BL‐ICs matched across frequency bands and the component demonstrated a consistent QRS‐like template.

### 2.7 Reconstruction Analysis

When projecting the BL‐ICs from the source space back into the signal space, the weights in the mixing matrix corresponding to the ECG artifacts were zeroed. Using the same scaler applied to the original band‐passed source space signals before ICA, we take the inverse transform to rescale the reconstructed signals back to the original signal space. This enabled reconstruction of the original signals without heart artifacts. After reconstruction without the ECG‐laden components, we conducted two further analyses. These analyses assessed how artifact removal enhanced the signal quality by decreasing interchannel similarity and increasing intrachannel differences.

A cross‐correlation analysis with zero lag was performed before and after BL‐ICA was applied to the signals, quantifying a reduction in shared noise across channels after the cleaning. This analysis evaluated only the rest periods of the motor mapping tasks, as motor attempts may alter the cross‐correlation coefficients between channels. After extracting the rest periods for a given run, we first normalize the signals for each channel and compute the cross‐correlation at zero lag. This normalization ensures we only get cross‐correlation coefficients that range between ‐1 and 1. Suppose we were to compute the value between the signals on two channels, *x* and *y*. We first normalize the signals by z‐scoring them to get $\tilde{x}=\frac{x-\mu_{x}}{\sigma_{x}}$ and $\tilde{y}=\frac{y-\mu_{y}}{\sigma_{y}}$. Then, we can compute the cross‐correlation coefficients as follows:

$$R_{xy}\left(0\right)=\left(\tilde{x}*\tilde{y}\right)\;\left(1-\left|\left|\tilde{x}\right|\right|\right)$$

where ||*x*|| was the length of the signal *x*, ||*x*|| = ||*y*||, and * denotes a discrete linear convolution. The correlation matrix was computed for each combination of channels for each run, then averaged to find the average cross‐correlation values during rest across all sessions. This analysis was performed in each of the alpha, beta, and low gamma bands, both before and after BL‐ICA was applied.

To determine the difference between the vECoG signals during the rest and go periods, we computed the relative entropy of the signals for each channel. Relative entropy evaluates the difference in uncertainty between the joint entropy of two distributions and the marginal entropy of one of the distributions. In other words, it was a measure of the distance between two distributions [49]. For our rest and go period relative entropy analysis, this can be written as:

$$H_{R}\left(P_{rest}\left|\right|P_{go}\right)=H\left(P_{rest},\;P_{go}\right)-H\left(P_{rest}\right)$$

where *H_R_* was the relative entropy, *P_rest_* was the distribution of rest signal amplitudes and *P_go_* was the distribution of the go signal amplitudes. We can rewrite this equation as follows:

$$H_{R}\left(P_{rest}\left|\right|P_{go}\right)=\int_{x}^{\;P_{rest}}\left(x\right)log_{2}\frac{P_{rest}\left(x\right)}{P_{go}\left(x\right)}d\left(x\right)$$

In this case, we apply relative entropy to assess how different the rest and go vECoG signal distributions were. Since the relative entropy quantifies how distinguishable the rest and go amplitude distributions were, it serves as a model‐agnostic proxy for the class separability that a decoder exploits; an increase after artifact removal indicates that the two task states were more readily discriminated. Probability distributions *P_rest_* and *P_go_* were estimated using histograms with a constant range and bin size. The envelope of rest and go period signals were found using the Hilbert transform method described in 2.5 prior to computing the distributions.

### 2.8 Beta Burst Detection

Recent work on the COMMAND EFS clinical trial has shown that low gamma and high gamma were more differentiable during rest and go periods than beta band [50], despite previous work in the Australian SWITCH trial using beta bursts as a motor feature [23]. We hypothesized that this may be the result of using different referencing schemes, as the analysis in [50] used a local reference and the analysis in [23] used a distal reference, and wanted to verify that beta bursts could be preserved with our proposed BL‐ICA cleaning method. Bursts were detected in beta band using the Bycycle Python package [51], which evaluates a time series on a cycle‐by‐cycle basis. The parameters for beta burst detection were set as follows: an amplitude fraction threshold of 0.6, an amplitude consistency threshold of 0.3, a period consistency threshold of 0.5, a monotonicity threshold of 0.6 and a minimum of 3 cycles per detected burst. The values for the amplitude consistency, period consistency and monotonicity thresholds were based on those used in the motor cortical beta oscillation analysis in [51]. Beta bursts have been shown to last for approximately 150 ms, which was approximately three beta cycles (≈50 ms each) [52, 53, 54]. This informed our decision to threshold burst detection to a minimum of three cycles.

## 3 Results

We are presenting preliminary results from six participants (P1‐P6) with severe paralysis who were implanted with a Stentrode array in the SSS adjacent to the primary motor cortex (Figure 1B). Based on data availability, Section 3.2 focuses on five of the participants, as P2 only had baseline recordings with a distal reference. Consequently, in Section 3.3 and 3.4, we focus on results from P1 and P6, which are the only participants with distally referenced motor mapping data suitable for a rest‐versus‐go analysis. Although local and distal referencing options were available, the local reference configuration was generally favored for real‐time BCI control tasks, which were the primary focus of the in‐home testing. Thus, the majority of testing sessions were performed with the system configured for local referencing.

### 3.1 Traditional Referencing Schemes May Not Preserve Cortical Motor Features

**FIGURE 2:**
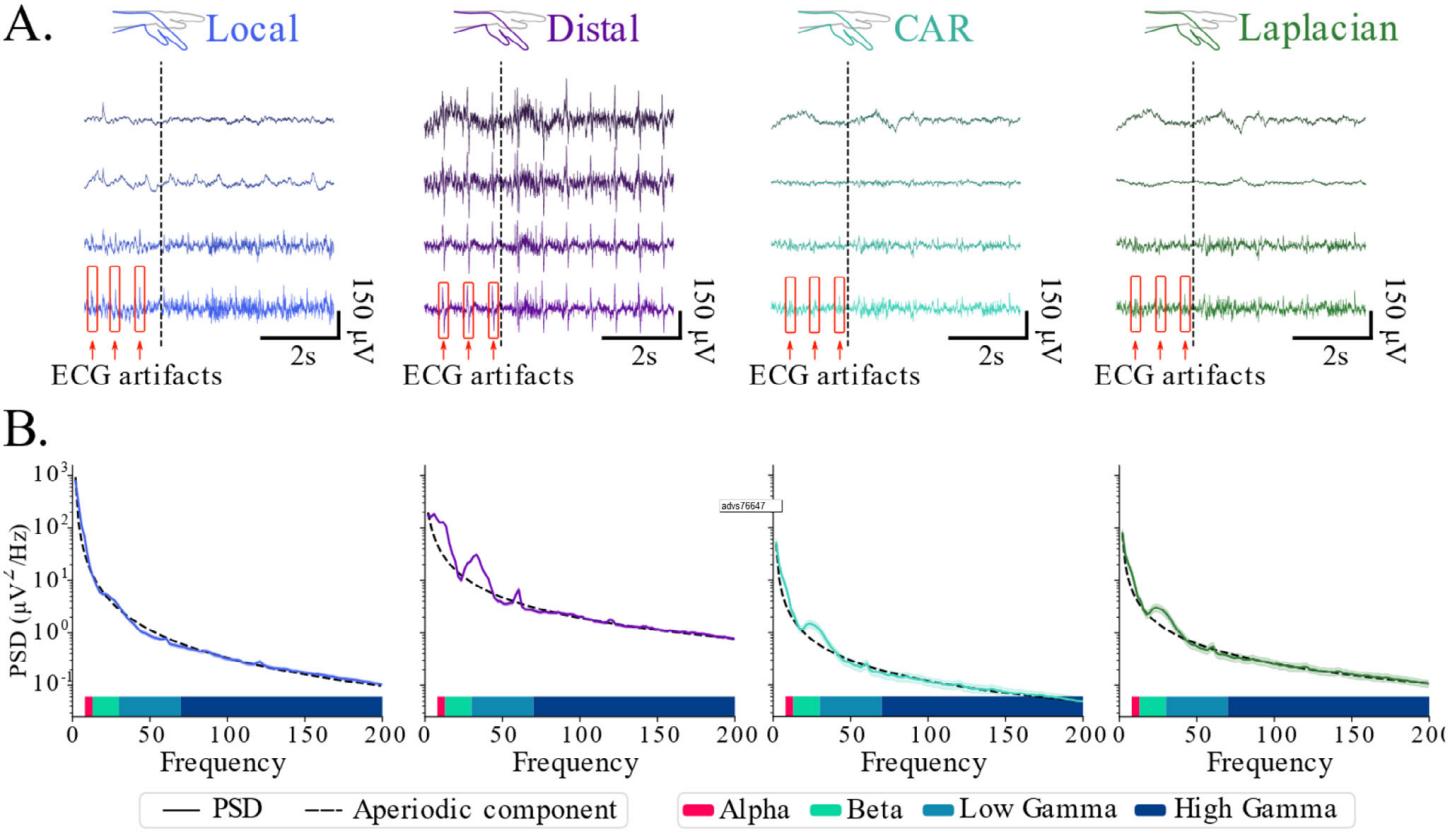
Referencing scheme signal quality for vECoG data. (A) Both online hardware referencing schemes and offline software re‐referencing. Sample channels are taken from P6 during the motor mapping task with attempted right hand movement. Three examples of preserved ECG artifacts are highlighted in red on one channel for each referencing scheme. Recall that the local referencing scheme is a hardware reference, and thus the time series depicted is a different run than the one depicted for Distal, CAR and Laplacian referencing. Thus, the timing of the ECG artifacts in the Local referencing scheme is not expected to align with those in the other three schemes. (B) Power spectral density plots of the baseline recordings for P6 across sessions. The black dotted curve on each axis is the estimated aperiodic component of the average baseline PSD using FOOOF [45]. The solid, colored line on each axis represents the average baseline PSD with the specific referencing scheme, across sessions and channels. Motor cortical frequency bands of interest are highlighted along the x‐axis.

To investigate whether typical cortical referencing schemes clean data at the expense of some motor features, we look at 4 schemes: local, distal, common average and laplacian referencing (Figure 2A). The latter two schemes were applied to data that was originally hardware distally referenced.

The primary source of noise when recording vECoG data comes from electrocardiogram (ECG) artifacts. While locally referenced data maintains some ECG artifact similar to previous work [33], both the time series (Figure 2A) and PSD (Figure 2B) of locally referenced data appear much more stable than that of the distally referenced data. To evaluate the persistence of ECG artifacts through re‐referencing methods, we detect ECG artifacts on a channel of the distally referenced data using BioSPPy [55] and use the relevant time points to evaluate the persistence of ECG artifacts after re‐referencing. For all participants, the local, CAR and Laplacian reference amplitudes were significantly different from distally referenced ECG artifact amplitudes (*p* < 0.001). While the alternative referencing schemes consistently reduce the amplitude of the ECG artifacts, whether they preserve low‐frequency motor‐related activity remains open.

To assess the impact of these referencing schemes on the preservation of low‐frequency motor features in the cortical recordings, we evaluate the presence of beta band in baseline recordings. Beta band activity is known to be higher at rest and inhibited during movement [56]. Lower frequency neural activity (such as alpha and beta bands) has a larger spatial spread than higher frequency activity (e.g. low and high gamma bands) [57, 58] and thus may have more similar power across electrodes as opposed to high frequency activity. This difference in spatial distribution indicates that lower frequency activity may be heavily impacted by referencing scheme choice. As noted in 2.8, a recent study analyzing locally referenced data from the COMMAND EFS trial did not see large differences in beta band between rest and go periods [50], while a previous Australian study using the distal reference used beta band modulation as their feature of choice [23]. Thus, we evaluate whether the four typical cortical referencing schemes capture baseline beta band activity.

At rest, beta band should have increased activity that presents as a bump in the PSD [58, 59]. As previously noted, during baseline recordings, our participants are instructed to relax their muscles and sit still for 2 minutes at a time. Though the Stentrode is recording bilaterally, we would expect to see similarly increased beta band activity across electrodes as compared to the 1/f frequency since there is no voluntary movement on either side. Thus, we should be able to average the PSDs across all channels for a representation of vECoG beta band activity during rest. In Figure 2B, these average PSDs are shown by the solid line for Distal, Local, CAR and Laplacian referencing schemes, respectively. The standard deviation of these PSDs is illustrated by the shading around the solid lines.

Distal, CAR and Laplacian referencing schemes appear to capture a larger amount of beta activity than the Local referencing scheme, as evidenced by the presence of clearer peaks in the average PSD for the non‐Local referencing schemes. Recall that the local and distal referencing schemes are hardware references, while the CAR and Laplacian are re‐referencing schemes applied to data originally collected with a distal referencing scheme. To ensure that we accommodate the difference in the aperiodic component of the signals that may be present due to the different sessions used and variation in referencing schemes, we estimate the aperiodic component of the average PSD using FOOOF [45] for each referencing scheme. These aperiodic components are illustrated in Figure 2B by the black dotted lines. Deviation from this dotted line can be interpreted as oscillatory activity. The same plots for all 6 included participants can be found in Figure S1. The average PSDs when using the Distal, CAR, or Laplacian referencing schemes show deviations from the estimated aperiodic components over the beta band range, whereas the Local referencing scheme does not. Furthermore, the Distal, CAR, and Laplacian schemes show deviations above the estimated aperiodic component, which indicates an increase in the oscillatory beta activity relative to other frequency bands during rest. The average and standard deviation of these oscillatory deviations from the fitted aperiodic component can be found in Table 2 for all 6 participants and 4 referencing schemes. These results indicate that the Local referencing scheme may be sacrificing some lower frequency activity while maintaining some of the ECG artifact. We note that the resting PSD deviation alone cannot distinguish genuine beta oscillations from beta‐range spectral energy contributed by the ECG artifact. We address this attribution directly using BL‐ICA (Sections 3.3–3.4; see Discussion).

**TABLE 2:** There is a tradeoff between reducing the ECG artifact and preserving resting beta oscillatory activity. For each participant and referencing scheme, we calculate two values. A_ECG_ (%) is the percentage reduction in ECG artifact amplitude from the distally referenced data in the format mean (standard deviation). d_β_ (µV^2^/Hz) is the average distance between the baseline PSD and the estimated aperiodic component for each referencing scheme.

ID	A_ECG_ (%)				d_β_ (µV^2^/Hz)			
	Local	Distal	CAR	Laplacian	Local	Distal	CAR	Laplacian
P1	−23.57 (66.18)	0.00 (0.00)	−38.34 (49.33)	−22.79 (67.44)	0.083	8.543	0.1216	0.1431
P2	−11.69 (137.46)	0.00 (0.00)	−44.69 (55.63)	−17.96 (119.09)	0.1900	79.505	2.9751	5.7428
P3	−84.32 (18.73)	0.00 (0.00)	−72.52 (26.15)	−59.21 (66.18)	1.2162	155.1197	18.1079	27.5673
P4	−88.14 (14.00)	0.00 (0.00)	−87.80 (14.19)	−83.85 (21.49)	22.1033	117.5132	14.7977	21.3571
P5	−84.32 (13.47)	0.00 (0.00)	−87.57 (11.16)	−68.05 (49.39)	0.4075	21.959	−0.169	3.367
P6	−87.31 (14.02)	0.00 (0.00)	−87.72 (8.87)	−83.47 (12.82)	−0.0974	17.544	0.422	0.862

### 3.2 Noise Separation through Blind‐Source Separation

**FIGURE 3:**
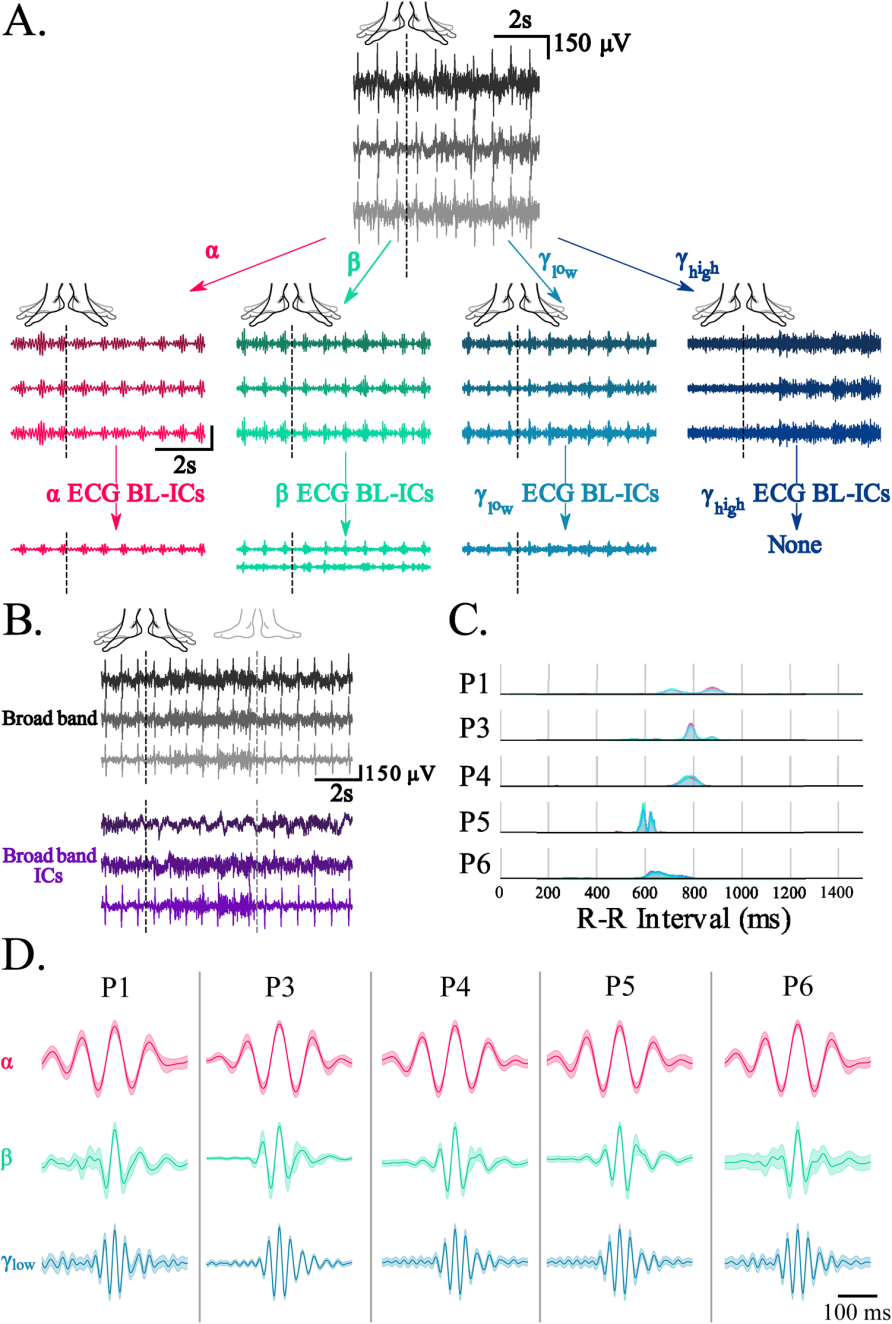
Band‐Limited Independent Component Analysis applied to vECoG data from P6. (A) The pipeline from raw vECoG data collected by the Stentrode to band‐limited independent components (BL‐ICs). The data shown is for bilateral ankle motor attempts in P6 with a distal monopolar reference which is subsequently band‐passed into alpha, beta, low gamma and high gamma bands. A band‐specific unmixing matrix is then constructed using FastICA [47], which reveals at least one component containing the electrocardiogram (ECG) artifact in the source space for alpha, beta and low gamma. (B) Example signals of distally referenced data (black) for bilateral ankle movements accompanied by several independent components resulting from ICA applied to the broadband signal. The task‐modulated signal is found on the independent component that captures the strongest ECG artifact, indicating broadband ICA may not be the best denoising technique. (C) The distribution of detected R‐R intervals on the isolated ECG components for all 5 participants. R‐R intervals were calculated using [48]. (D) The average detected ECG waveform for each participant, averaged across all trials and all ECG BLICs in alpha, beta and low gamma bands. Waveform templates were found using [48].

Typical referencing schemes explored in 3.1 greatly reduce, but do not eliminate, ECG artifacts in the vECoG data. As previously mentioned, ICA and other blind source separation techniques have become commonplace when removing artifacts from non‐invasive neural recording techniques [60]. A prior investigation demonstrated that ICA can be leveraged on the Stentrode to achieve a greater reduction of ECG artifacts than using the local referencing scheme [33]. However, when performing ICA on broadband data, we do not consistently see clear separation of ECG from the neural signals (Figure 3B). This may occur due to the large number of sources, both neural and noise, that are contributing to the broadband power spectra – with only 9–13 electrodes, it may not be feasible to identify and separate all sources in that signal. Instead, we propose using BL‐ICA, which leverages ICA's proven prowess for identifying ECG artifacts by limiting the sources contributing to the input signal, in hopes that this may facilitate more accurate source space decomposition of the vECoG signal.

To determine the number of components used for our BL‐ICA analysis, as described in the Methods section, we performed FastICA on all motor mapping task runs with all possible numbers of components, upper bounded by the number of channels being used for analysis, and computed the L2 norm of the reconstruction error for each run in each band. All five participants had the lowest reconstruction error for each band when applying BL‐ICA with a number of components equal to the number of good channels on their implant (Table 3, Figure S2). For each band in each participant, the reconstruction error was nearly zero when using the maximum number of components.

**TABLE 3:** Reconstruction error. For each participant, the best number of components to reconstruct the data was the maximum number of components. Here, we have listed the reconstruction error across all the distally referenced data for each band, in the format median ± standard deviation. The reconstruction error is calculated as the L2 norm of the difference between the observed band‐passed signals and the signals reconstructed after ICA.

Participant ID	Number of components	Alpha	Beta	Low Gamma	High Gamma
P1	13	0.243 ± 0.041	0.008 ± 0.015	0.003 ± 0.001	0.002 ± 0.002
P3	12	0.055 ± 0.033	0.090 ± 0.056	0.005 ± 0.001	0.001 ± 0.000
P4	11	0.034 ± 0.053	0.025 ± 0.094	0.004 ± 0.003	0.003 ± 0.002
P5	10	0.011 ± 0.000	0.020 ± 0.003	0.030 ± 0.040	0.002 ± 0.001
P6	9	0.005 ± 0.005	0.007 ± 0.006	0.007 ± 0.010	0.006 ± 0.003

After projecting the motor mapping task into the band‐limited source spaces via BL‐ICA, one to two ECG‐laden components appear in each of the alpha, beta, and low gamma bands (Figure 3A). The number removed per participant and band is reported in Table S1. BL‐ICA applied to high gamma band activity does not reliably isolate an ECG component; however, the time series of the band‐passed high gamma signal is often not heavily artifacted with ECG, likely due to its high frequency range (70–200 Hz) [28]. All 5 participants exhibit this separation of ECG in the alpha, beta, and low gamma bands.

To validate that we are indeed separating BL‐ICs containing ECG artifacts, we use BioSPPY to detect the artifacts on the identified ECG BL‐ICs, compute the R‐R intervals and find the template ECG waveform for the band [48]. This analysis was performed participant‐wise across all motor mapping sessions in the alpha, beta and low gamma bands. An R‐R interval of 800 ms corresponds with a heart rate of 75 beats per minute. For each participant, the R‐R intervals were in the range of normal heart rate across frequency bands (Figure 3C), suggesting that we are indeed properly identifying and separating ECG artifacts. The template ECG artifact waveform for each band is included for five participants, along with their standard deviation (Figure 3D).

### 3.3 Source Space Appears to Preserve Task‐Modulated Motor Features

With a focus on finding independent sources within a cortical frequency band, the question arises of whether or not we are able to separate more than just noise. Specifically, can we identify task‐modulated activity in the cortical bands of interest by using BL‐ICA? For this analysis, due to limited distally referenced data, we look only at P1 and P6 due to the availability of distally referenced motor data suitable for this analysis (see Sections 3 and 4.4).

**FIGURE 4:**
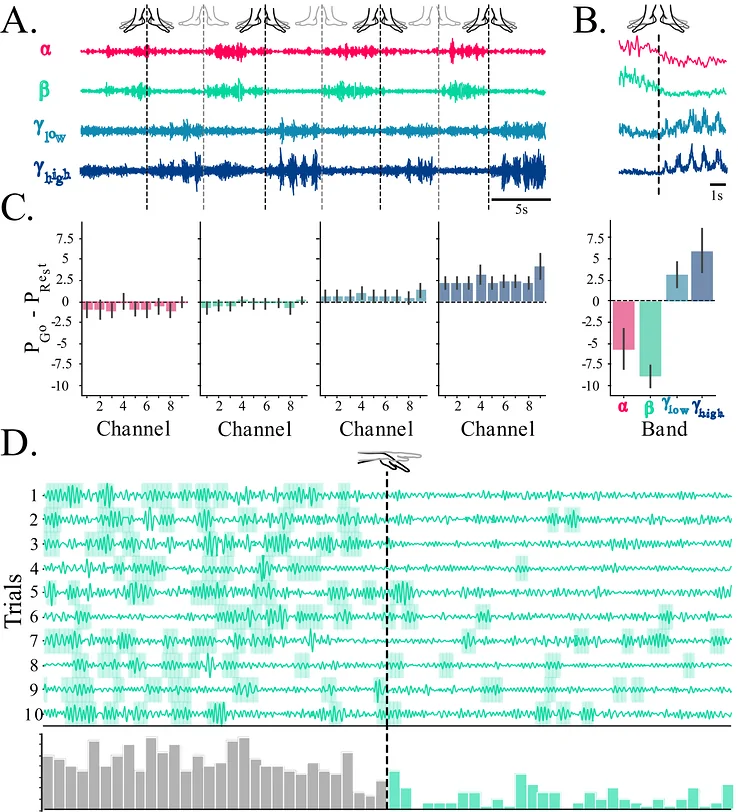
Task‐modulated independent components in the BL‐ICA source space. (A) The top task‐modulated BL‐IC for each cortical band of interest is shown as a time series during bilateral ankle movement in P6. (B) The average envelope of the top task‐modulated BL‐IC for the same run. (C) Difference in average power during rest and go trials for the run shown in (A, B). The four plots on the left visualize the power difference for the observed signals in alpha, beta, low gamma and high gamma for each channel. The fifth plot, on the right, shows the same calculation performed on the task‐modulated BL‐ICs. (D) beta burst detection on the top beta task‐modulated BL‐IC for bilateral ankle movement. Beta bursts are detected using the methodology explained in Section 2.8 in the source space. There is a clear decrease in beta burst frequency with the ‘Go’ cue, indicating that our task modulated beta BL‐IC not only preserves Event Related Desynchronization, but also the bursts relevant to bilateral ankle control.

In the source spaces of P1 and P6, a putatively task‐modulated BL‐IC can be identified in each cortical frequency band for each run. The timeseries of these BL‐ICs is consistent with a reduction (alpha and beta) or an increase (low/high gamma) in activity after the presentation of the go cue (Figure 4A). When looking at the envelopes of these putatively task‐modulated BL‐ICs, there are 5 distinct peaks in the low gamma and high gamma bands after the go cue is presented, which appear to correspond to the 5 attempted movements. In the alpha and beta band envelopes, there is a sustained depression during the go period, which may indicate that task‐modulated desynchronization is captured (Figure 4B).

To confirm there is a quantifiable change in the signal quality from the observed signal space to the BL‐ICA source space, we analyze the SNR between the rest and go periods for the original, band‐passed signals on each channel. As described in Section 2.5, this is equivalent to converting the power to decibels during rest and go in each band, and subsequently taking the difference. A negative value indicates desynchronization (higher power during the rest period) while a positive value indicates synchronization. Across most movements, there is muted desynchronization during the go periods for the alpha and beta bands, while low gamma and high gamma demonstrate synchronization. However, for all movement types, the top alpha and beta BL‐ICs are more strongly desynchronized than the individual band‐passed channels. (Figure 4C and Figure S3).

Within the top task‐modulated beta BL‐IC, beta bursting activity was preserved. As defined in Section 2.8, beta bursts were evaluated on a cycle‐by‐cycle basis. We expect to see more bursts during rest periods, and fewer bursts during active movement periods (after the go cue). We find that this clear relationship between bursting activity and movement attempts was maintained throughout the BL‐ICA decomposition process, and that large changes in beta bursting activity occur within the BL‐IC (Figure 4D).

### 3.4 Reconstruction Reduces Shared Noise and Improves Rest/Go Separability

**FIGURE 5:**
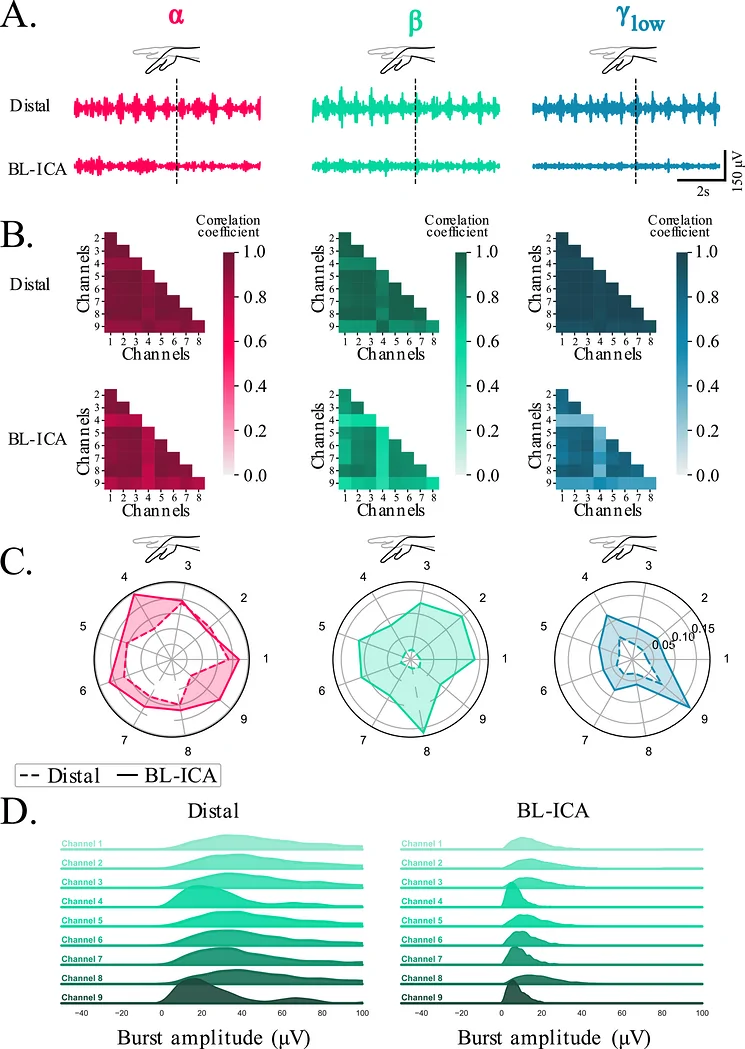
Reconstruction and somatotopy. (A) Example time series of a channel in alpha (α), beta (β) and low gamma (𝛾_low_) bands before and after ECG removal with BL‐ICA. (B) Cross‐correlation analysis of channels before and after ECG artifact removal with BL‐ICA across all sessions. The normalized cross correlation of the channels was computed for the rest periods of each run in alpha, beta and low gamma, both before and after BL‐ICA. These correlation matrices were then averaged across all runs. (C) Channelwise relative entropy measurements in the alpha, beta and low gamma bands before and after cleaning with BL‐ICA. The dotted line represents relative entropy on each channel for the band‐passed, distally referenced signals. The colored points represent the same data after ECG components are removed through BL‐ICA. (D) The distribution of detected beta band burst amplitudes after BL‐ICA reconstruction.

We first verify that ECG is acceptably removed from the signals after reconstruction without the ECG components. In each band, the timeseries before and after cleaning (right hand movement illustrated in Figure 5A) verify a large reduction in the amplitude of the observed signals. Additionally, we compute the zero‐lag cross‐correlation of each band before and after cleaning with BL‐ICA during rest. For most bands and nearly all participants, the channels become less correlated (Figure 5B and Figures S4 and S5). These results indicate that we are effectively removing a common source of noise, ECG, across the channels of the Stentrode.

To evaluate if BL‐ICA is actually improving discriminability between rest and go periods in the signals, we use relative entropy to assess the difference between the probability distributions of the rest and go signals, *H_R_* (*P_rest_* ||*P_go_*), on each channel. We perform this analysis both on the distally referenced signal and on the signal that is reconstructed after removing ECG components with BL‐ICA. Figure 5C illustrates the relative entropy in both cases for each frequency band on the same trial used in Figure 4A–C. The band‐passed, distally referenced relative entropy for each band is illustrated with a dotted line, while the BL‐ICA relative entropy values are illustrated by a solid line. The difference between the two values is shaded. A visualization of *H_R_* (*P_rest_* ||*P_go_*) for each of the 6 movement types for P6 can be found in Figure S6. In most cases, cleaning via BL‐ICA increases the *H_R_* (*P_rest_* ||*P_go_*) as compared to the bandpassed, distally referenced quantities. This indicates that, in the participants analyzed, BL‐ICA improves the discriminability of the probability distributions of the signal during rest and go periods by reducing common sources of noise present in both the rest and go distributions.

After reconstructing the signals with BL‐ICA cleaning, we also assess the amplitude of the detected beta bursts on signals in the observation space (original and reconstructed). Of note, there was a large downward shift in the distribution of detected beta burst amplitudes after cleaning (Figure 5D). However, the amplitude before cleaning was much higher than what would be expected for a real beta burst. Removing ECG artifacts with BL‐ICA drops the detected amplitudes into an expected range for beta burst, indicating that we may be uncovering true beta bursting activity that was masked in the observed signal.

## 4 Discussion

The results of this study indicate that ECG artifacts can be effectively removed from vECoG signals while maintaining beta‐band motor features in the participants examined by first band‐limiting the neural recordings, then fitting ICA to separate the noise source. Of the 6 participants implanted in the COMMAND EFS clinical trial, only 5 had movement data collected with a distal reference. All 6 participants had baseline data available with a distal reference. Since P2 only had locally referenced movement data collected, their data was not included in this study beyond Section 3.1.

### 4.1 Interpreting the Performance of Typical Referencing Schemes

Common average referencing (CAR) has been shown to be optimal for a microelectrode array given that 1) channel placement is sufficiently large and 2) the noise model for each channel is identical [61]. For brevity, we refer the reader to the original work for the proof. While previous work on the Stentrode has utilized a CAR scheme [41, 42, 43], Stentrode vECoG recordings violate the second assumption. The channels on the Stentrode form a cylindrical pattern along the sagittal plane of the brain, meaning some contacts may not face the cortex and thus will have different noise models. Additionally, since the Stentrode sits in the SSS between the hemispheres, it is not fair to assume that the noise model is identical across all electrodes.

Laplacian referencing, a local version of CAR, depends upon the availability of channels, their locations and their signal quality. The geometry of the Stentrode, combined with the placement between the two hemispheres, makes it difficult to take advantage of the benefits of the Laplacian referencing scheme. Located in the SSS, each side of the Stentrode is recording from a separate neural population, containing different noise and information content than the other side.

Local referencing appears to have a larger impact on the low‐frequency vECoG activity (alpha and beta bands) than high‐frequency activity (low gamma and high gamma bands). Low‐frequency activity is known to have a larger spatial spread than high‐frequency neural activity [62, 63]. In a monopolar referencing scheme, where all channels are close together, it follows that some shared low‐frequency activity may be removed erroneously along with common noise, without notable impact on the information contained in the higher‐frequency bands.

### 4.2 Distal Referencing with BL‐ICA Facilitates ECG Separation

While a distal monopolar reference appears to obscure the vECoG recordings with higher noise levels, the presence of a stronger noise source actually enables better separation of ECG from neural sources. When local referencing is performed, the amplitude of these artifacts are greatly reduced, which makes them more difficult to separate from the actual information contained in the neural signals. Thus, a more distal reference than a stent‐mounted channel is preferred for ECG removal via BL‐ICA.

Additionally, band‐limiting the neural signals ahead of performing ICA is more effective at separating ECG artifacts than using the broadband signal. ICA is limited in the number of sources it can isolate in part by the number of channels being recorded from – with a broadband signal, we are capturing neural sources in various frequency bands. These sources likely far outnumber the available vECoG channels, making it difficult to separate motor‐related neural signals from ECG artifacts. When the signals are band‐limited ahead of ICA, the number of input sources are reduced, enabling better identification and separation of independent components within the vECoG signals.

The choice of reference location and its impact on signal properties has similarly been explored in EEG. Selection of a monopolar reference in different locations across the scalp has been shown to impact the observed functional connectivity patterns [64].

### 4.3 Performance of BL‐ICA

The results presented in this study demonstrate clear ECG separation from neural signals when BL‐ICA is applied to vECoG signals. ECG‐laden components were verified using R‐R interval detection and analysis. Previous work has shown ALS participants have shorter R‐R intervals than healthy participants [65, 66]. In fact, sinus tachycardia, which is defined as R‐R intervals of 400–600 ms or 100–150 beats per minute, was found to be the most common type of arrhythmia found in ALS patients [66, 67]. Two of our participants, P5 and P6 had slightly faster heart rates (Figure 3C), and this can likely be attributed to their prognosis.

After removing the ECG BL‐ICs, cross‐channel correlation was shown to decrease for alpha, beta and low gamma bands. This indicates that a common signal across all channels was removed from the signals, both during rest and movement conditions. Additionally, a general increase in relative entropy between rest and go conditions was observed, indicating that the distribution of neural activity on most channels and in most frequency bands was more differentiable after applying BL‐ICA. Most notably, beta desynchronization and bursting activity was preserved after removing the ECG BL‐ICs. Together, these results suggest that BL‐ICA applied to a distally referenced vECoG signal likely separates neural signals from ECG artifacts. A more distal reference may preserve neural features better while facilitating artifact removal than a brain‐to‐brain, or local, referencing scheme. If a hardware monopolar reference is desired, then placement of a reference electrode more proximal to the brain than the IRTU, but not located directly on the stent body, should be considered.

### 4.4 Limitations and Future Directions

Several limitations should be noted. First, the analyses are restricted to the participants and movement types for which distally referenced data were available. The COMMAND EFS is an early feasibility study, and the test protocol favored local referencing over distal referencing. Thus, the amount of distally referenced recordings differs across participants. Consequently, the consistency of ECG separation was demonstrated across the five participants with distally referenced motor data (Figure 3, Table 3), while the more detailed analyses of task‐modulated source‐space features and somatotopy were limited to P1 and P6. These latter analyses are intended as demonstrations that known motor features (beta desynchronization, beta bursting, gamma synchronization) are preserved through BL‐ICA cleaning, validated against well‐established motor physiology, rather than as population‐level statistical claims. Broader validation across additional participants will be needed to establish generalizability as more distally referenced data become available.

A second limitation of this study is that the BL‐ICA denoising methods were evaluated with offline measures, because the clinical trial protocol did not allow for testing of the BL‐ICA denoising methods in real‐time. Instead, we used two metrics that relate directly to functional utility. The relative entropy between the rest and go distributions measures the separability of the two task states, which is the property that ultimately governs decodability of the states. This metric increased after BL‐ICA cleaning across most channels and bands, reflecting greater discriminability between rest and go states. The preservation of beta‐band desynchronization and bursting (Figures 4D, 5D) is likewise functionally relevant, as beta modulation served as the control feature in an earlier human Stentrode study [23]. A direct comparison of decoding performance across referencing and artifact‐removal methods is an important next step.

A potential concern is whether the larger beta‐range power under distal referencing reflects genuine neural oscillations or residual ECG energy in the beta band, given that the distal reference retains the most cardiac artifact and that ECG can alter the spectral content of neural recordings [27]. Several lines of evidence argue against the preserved beta being purely cardiac in origin. BL‐ICA identifies the ECG component by its characteristic R‐R interval periodicity and QRS template waveform (Figure 3C, D), which is specific to cardiac activity. The beta activity we report as preserved resides on separate components that lack this periodicity and instead desynchronize time‐locked to the go cue (Figure 4A, B). This is a modulation that cardiac artifacts cannot produce, as it is locked to the cardiac cycle rather than the task. Additionally, detected beta burst amplitudes fall into the physiologically expected range only after the ECG component is removed (Figure 5D). The inflated amplitudes in the distally referenced data before BL‐ICA cleaning confirm that ECG artifact did contribute to the apparent beta power, but the persistence of bursting at physiological amplitudes after ECG removal indicates an underlying genuine beta component. We therefore interpret the resting PSD comparison (Section 3.1) as motivating the tradeoff between ECG suppression and beta‐range power, with the BL‐ICA analyses providing the means to attribute the preserved beta to neuromotor rather than cardiac sources.

## Author Contributions

**D.W**., **D.P**., **T.O**., **J.C**., **N.O**., **R.N**., **N.H**., **D.L**., **A.F**., **P.Y**., and **J.B**. conceived the study. **D.W**., **D.P**., **T.O**., and **N.O**. secured funding. **N.H**. and **D.L**. coordinated clinical management. **R.N**. and **S.M**. performed the implantation surgery. **A.K.F**. led the data analysis with assistance from **K.K**., **L.Y**., **N.C**., **D.W**., **P.Y**., **J.B**., and **J.C. A.K.F**. wrote the paper and all authors contributed to editing.

## Grants

The work presented in this study was supported by NIH Grant UH3NS120191.

## Conflicts of Interest

JB, PY, TO, NO, and AF are employees at Synchron Inc. and hold stock options. TO and NO are the founding directors and shareholders of Synchron Inc. TO and NO have a patent for the Stentrode (US 10,485,968 B2). DW is a cofounder and shareholder of ReachNeuro and holds stock options from NeuroOne and NeuronOff. A.K.F., K.K., L.Y., N.C., N.H., D.L., R.N., J.C. and D.P. have no conflicts of interest to report.

## Supporting information




**Supporting File 1**: advs76647‐sup‐0001‐SuppMat.docx.

**Supporting File 2**: advs76647‐sup‐0002‐FigureS1‐S5.zip.

## Data Availability

The data that support the findings of this study are available from the corresponding author upon reasonable request.
