# Supplementary material for: Preserving Motor Features by Alternative Re‐Referencing to Remove Heart Artifact on the Stentrode
Source: Adv Sci (Weinh). 2026 Sep 4;13(56):e76647. doi: 10.1002/advs.76647 (PMC13544191; doi:10.1002/advs.76647)
Supplement: Supplementary file 1 [file ADVS-13-e76647-s002.docx]

Supporting Information

**Preserving motor features by alternative re-referencing to remove heart artifact on the Stentrode**

Ariel K. Feldman^1,2^, Kriti Kacker^3^, Rahyun Yun^4^, Nikole Chetty^3^, James Bennett^5,6^, Peter E. Yoo^5,6^, Raul G. Nogueira^8^, Jennifer L. Collinger^9,10,11,12^, Nicholas L. Opie^5,6^, Thomas J. Oxley^5,6^, David F. Putrino^7^ and Douglas J. Weber^1,2,3^

^1^ Neuroscience Institute, Carnegie Mellon University, Pittsburgh, PA, USA

^2^ Center for the Neural Basis of Cognition, Pittsburgh, PA, USA

^3^ Department of Mechanical Engineering, Carnegie Mellon University, Pittsburgh, PA, USA

^4^ Department of Electrical & Computer Engineering, Carnegie Mellon University, Pittsburgh, PA, USA

^5^ Vascular Bionics Laboratory, Department of Medicine, Royal Melbourne Hospital, University of Melbourne, Parkville, Victoria, Australia

^6^ Synchron, Inc., New York, NY, USA

^7^ Department of Rehabilitation and Human Performance, Icahn School of Medicine at Mount Sinai, New York, NY, USA

^8^ Department of Neurology and Neurosurgery, University of Pittsburgh Medical Center, Stroke Institute, Pittsburgh, Pennsylvania

^9^ Rehab Neural Engineering Labs, University of Pittsburgh, Pittsburgh, PA, USA

^10^ Department of Physical Medicine and Rehabilitation, University of Pittsburgh, Pittsburgh, PA, USA

^11^ Department of Bioengineering, University of Pittsburgh, Pittsburgh, PA, USA

^12^ Department of Biomedical Engineering, Carnegie Mellon University, Pittsburgh, PA, USA

***Supplementary Figure 1.*** ***Baseline power spectra show distal reference preserves low frequency activity most consistently.*** *The baseline power spectral density of all 6 participants included in this study for local reference (dark blue, left), distal reference (purple, middle left), common average re-reference (turquoise, middle right) and Laplacian re-reference (green, right). The dotted lines are the FOOOF* [*(42)*](https://paperpile.com/c/kBxyFY/hLnI) *fit of the aperiodic component of each PSD. Each PSD is plotted in log-log scale.*

***Supplementary Figure 2.*** ***Parameter selection for Independent Component Analysis.*** *L2 norm of reconstruction errors for P1 and P3 – P6 when performing BL-ICA on distally referenced data (****A-E****).*

| Participant ID | Decomposition size | Alpha ECG | Beta ECG | Low Gamma ECG | Total duration (s) |
| --- | --- | --- | --- | --- | --- |
| P1 | 13 | 1.67 (0.516) | 2.00 (0.00) | 1.00 (0.00) | 619.9 |
| P3 | 12 | 2.00 (0.00) | 2.00 (0.00) | 2.00 (0.00) | 218.0 |
| P4 | 11 | 1.33 (0.577) | 1.33 (0.577) | 1.67 (1.16) | 323.7 |
| P5 | 10 | 1.00 (0.00) | 2.00 (0.00) | 1.50 (0.71) | 211.2 |
| P6 | 9 | 1.00 (0.00) | 2.29 (0.54) | 1.07 (0.26) | 2772.7 |

***Supplementary Table 1. Average number of ECG components removed.*** *For each participant, the total number of components used for BL-ICA decomposition is listed, along with the number of ECG components detected and removed in each band for a given trial, in the format mean (standard deviation). Additionally, the total duration of the distally referenced motor mapping tasks included in the analysis is listed in seconds.*

***Supplementary Figure 3.*** ***SNR for band-passed vs BL-ICA source space cleaned signals.*** *Left four columns indicate band-passed SNR, far right column indicates the top task-modulated BL-IC SNR. The top six rows correspond with each movement for P6, the bottom row corresponds with bilateral ankle movements for P1.*

***Supplementary Figure 4.*** ***Correlation before and after BL-ICA during rest.*** *Zero lag correlation of Stentrode channels before and after ECG artifact is removed with BL-ICA.*

***Supplementary Figure 5.*** ***Correlation before and after BL-ICA during movement.*** *Zero lag correlation of Stentrode channels before and after ECG artifact is removed with BL-ICA. All movement types collected (six for P6 and one for P1) are included, as they have the most distally referenced data.*

***Supplementary Figure 6.*** ***BL-ICA generally improves relative entropy.*** *Each row indicates a movement type, and each column indicates a frequency band. The dotted line depicts the values of* $H_{R}(P_{rest}||P_{go})$ *for the distally referenced data, while the solid line shows* $H_{R}(P_{rest}||P_{go})$ *after BL-ICA. The shading highlights the difference between the distal and BL-ICA re-referenced data.*
